# Product Speculation from Carotenogenic Gene Cluster of *Nonlabens spongiae* Genome, and Identification of Myxol and Functional Analysis of Each Gene

**DOI:** 10.3390/genes16020202

**Published:** 2025-02-07

**Authors:** Keisuke Nakazawa, Daiki Mineo, Takuya Harayama, Susumu Yoshizawa, Shinichi Takaichi, Kenjiro Sugiyama

**Affiliations:** 1 Department of Applied Chemistry, School of Advanced Engineering, Kogakuin University, Nakanomachi, Hachioji 192-0015, Tokyo, Japan; bm18034@g.kogakuin.jp (K.N.); s219080@g.kogakuin.jp (D.M.); s218070@g.kogakuin.jp (T.H.); 2 Graduate School of Frontier Sciences, The University of Tokyo, Kashiwa 277-8563, Chiba, Japan; yoshizawa@aori.u-tokyo.ac.jp; 3 Atmosphere and Ocean Research Institute, The University of Tokyo, Kashiwa 277-8564, Chiba, Japan; 4 Department of Molecular Microbiology, Faculty of Life Sciences, Tokyo University of Agriculture, Sakuragaoka, Setagaya 156-8502, Tokyo, Japan; shintakaichi@gmail.com

**Keywords:** carotenoid, flavobacteria, gene cluster, metabolic engineering, monocyclic carotenoid, product speculation

## Abstract

**Background:** Myxol, a monocyclic carotenoid with β- and ψ-end groups, has been identified in only a limited number of bacteria, such as flavobacteria and cyanobacteria. Despite its biological significance, the biosynthetic pathway of myxol is not well understood, and studies on its physiological functions and biological activities are limited because of its rarity. **Methods:** BLAST homology searches for carotenoid biosynthesis genes in the genome of *Nonlabens* were performed. The carotenogenesis-related genes in the genome of the marine flavobacteria *Nonlabens spongiae* were individually cloned and functionally characterized using a heterologous *Escherichia coli* expression system. Carotenoids from *N. spongiae* were identified using an LC-MS analysis. **Results:** We identified a gene cluster involved in carotenoid biosynthesis in the genome of *N. spongiae*. This cluster includes genes encoding phytoene synthase (CrtB), phytoene desaturase (CrtI), lycopene cyclase (CrtY), carotenoid 1,2-hydratase (CruF), carotenoid 3,4-desaturase (ψ-end group) (CrtD), carotenoid 2-hydroxylase (ψ-end group) (CrtA-OH), and carotene hydro-xylase (CrtZ). Based on the characteristics of these enzymes, the primary products were predicted to be myxol and/or zeaxanthin. A spectroscopic analysis confirmed that myxol was the primary carotenoid. Furthermore, a plasmid containing a reconstructed gene cluster and geranylgeranyl pyrophosphate synthase (CrtE) located outside the cluster was introduced into *E. coli*. This system predominantly accumulated myxol, indicating that the reconstructed gene cluster enabled efficient myxol production in *E. coli*. **Conclusions:** This study highlighted the potential biotechnological applications of the carotenoid biosynthesis gene clusters for myxol production.

## 1. Introduction

Carotenoids are isoprenoid pigments synthesized by all photosynthetic and several nonphotosynthetic organisms, such as bacteria, algae, fungi, and higher plants. They play significant roles in various biological functions, such as light harvesting, photoprotection, assembling pigment–protein complexes in phototrophs, and the stabilization of lipid membranes [1,2,3]. Carotenoids perform diverse functions that contribute to human health, including immune enhancement, exhibiting antioxidant activity, and serving as a precursor for vitamin A production. More than 850 structurally different carotenoids exist in nature [3,4] with their diversity resulting from variations in the carotenogenesis pathways, different characteristics of carotenogenic enzymes, and the specific genes involved in carotenogenesis.

After the identification of carotenoids, several carotenogenesis-related genes have been isolated from various organisms, characterizing their functions. Carotenogenesis gene clusters have been identified in several bacteria, including *Rhodobacter capsulatus* for spirilloxanthin [5,6] and *Pantoea ananatis* for zeaxanthin diglucoside [7]. All the carotenogenic genes in their clusters of these bacteria were isolated and functionally assigned. Furthermore, all purple bacteria possess a photosynthetic gene cluster, including carotenogenic genes for spirilloxanthin and spheroidenone [3]. The aerobic photosynthetic bacterium *Bradyrhizobium* spp. ORS278 has two sets of gene clusters, one for canthaxanthin and the other for spirilloxanthin biosynthesis [8,9]. In cyanobacteria, algae, fungi, and higher plants, the carotenoid biosynthesis genes do not form clusters. However, candidate carotenogenesis-related genes can be identified from the genomic DNA sequence using an in silico analysis and the products can be predicted based on the characteristics of the encoded proteins of these genes, such as in the approach for zeaxanthin from *Sphingomonas sabuli* [10] and bacterioruberin from haloarchaea [11].

Myxol, a monocyclic carotenoid with β- and ψ-end groups, is named after the aglycon of myxoxanthophyll, commonly found in cyanobacteria, such as *Oscillaria*, *Spirulina*, and *Synechocystis* sp. PCC 6803 [12,13,14]. Free myxol was first identified in the strain P99-3, an orange pigment-producing marine bacterium belonging to the family *Flavobacteriaceae* and isolated from a sponge in Palau [15]. Myxol possesses stronger antioxidant activity compared to β-carotene and zeaxanthin, which also have dicyclic structures. Despite its biological significance, the biosynthetic pathway of myxol is not well understood, and studies on its physiological functions and biological activities are limited.

Several genes involved in myxol synthesis have been studied in flavobacteria and cyanobacteria. In *Flavobacterium* sp. strain P99-3, functions of lycopene cyclase (CrtYm) [16], carotene hydroxylase (CrtZ) [17], carotenoid 3,4-desaturase (ψ-end group) (CrtD) [18], and carotenoid 2-hydroxylase (ψ-end group) (CrtA-OH) [19] were investigated. Similarly, in cyanobacteria, the CruF protein from *Synechococcus* sp. strain PCC 7002 was functionally identified as a carotenoid 1,2-hydratase [20]. However, the functions of some of these genes remain unknown.

Recent research has highlighted the efficient production of useful carotenoids such as lycopene, β-carotene, and zeaxanthin by metabolic engineering methods using heterologous *Escherichia coli* expression systems. These systems can produce farnesyl pyrophosphate (FPP) but lack the biosynthesis genes for carotenoids. Metabolic engineering has also been used to produce rare carotenoids and novel non-natural carotenoids [21,22,23].

*Nonlabens spongiae* JCM 13191^T^ belonging to the family *Flavobacteriaceae*, isolated from the marine sponge *Lissodendoryx isodictyalis* in the Bahamas, contains an orange pigment [24]. In this study, we found some carotenogenesis-related genes in the genome, located in a carotenogenesis gene cluster, using an in silico analysis. Based on the characteristics of the candidate genes, the products were predicted as either myxol and/or zeaxanthin. We then identified the products and functionally characterized each gene in the cluster. Furthermore, we succeeded in the heterologous production of myxol in *E. coli* using reconstructed genes from *N. spongiae*.

## 2. Materials and Methods

### 2.1. Genome and Gene Clusters from Nonlabens

Genomic DNA sequences of nine species of genus *Nonlabens* were retrieved from GenBank, including *N. spongiae* JCM 13191^T^ (GCA_002117125.1), *N. dokdonensis* DSW-6^T^ (GCA_000332115.1), *N. xylanidelens* DSM16809^T^ (GCA_002934445.1), *N. tegetincola* JCM 12886^T^ (GCA_002954355), *N. tegetincola* NBRC 100970 (previously *N. sediminis*) (CP019342), *N. arenilitoris* KCTC 32109^T^ (GCF_002954765.1), *N. ulvanivorans* PLR^T^ (GCF_000732625.1), *N. agnitus* JCM 17109^T^ (GCF_002994045.1), and *N. marinus* S1-08^T^ (GCA_000831385.1) [24,25,26,27,28,29,30,31,32,33].

To identify the carotenogenic gene clusters in *Nonlabens*, BLAST homology searches for *crtB*, *crtI*, *crtY*, *cruF*, *crtD*, *crtA-OH*, and *crtZ* were performed on the genomic sequences of the nine *Nonlabens* species, based on previous reports [16].

### 2.2. Strains and Growth Conditions

*Nonlabens spongiae* JCM 13191^T^ was provided by the Japan Collection of Microorganisms (JCM) [24,25]. The strains were cultured on a marine broth medium (Becton, Dickinson and Company, Franklin Lakes, NJ, USA) at 25 °C.

### 2.3. Extraction and Analysis of Carotenoids from N. spongiae

*N. spongiae* cells were centrifuged (15,000 rpm, 1 min), and pigments were extracted with acetone/methanol (7:2, *v*/*v*) using ultrasonication (two times of 20 s). After centrifugation (15,000 rpm, 5 min), the supernatant was collected and dried under N_2_ flow, and the dried residue was dissolved in methyl tert-butyl ether/methanol (7:3, *v*/*v*). HPLC was performed using a Vanquish Series with a photodiode array detector (Thermo Fisher Scientific, Waltham, MA, USA). A C30 YMC column (250 × 4.6 mm, 5 μm) (YMC America, Inc., Devens, MA, USA) was employed for the separation. The extract was eluted at a rate of 0.8 mL min^−1^ with solvent A (water/methanol, 5:95, *v*/*v*) for 2 min, followed by a linear gradient from solvent A to solvent B (methyl tert-butyl ether/methanol, 7:3, *v*/*v*) for 23 min and solvent B alone for 15 min. An Orbitrap Exploris 120 Mass Spectrometer (Thermo Fisher Scientific) with atmospheric-pressure chemical ionization (APCI) was used for a mass analysis. The capillary temperature was set to 250 °C, and the APCI vaporizer temperature was maintained at 400 °C. Screening was performed in a full scan mode, covering the range of *m*/*z* 100–1000. Carotenoids were identified based on their HPLC retention time and their characteristic absorbance spectra in the eluent as well as mass spectra, and compared with those of synthesized myxol [34] and zeaxanthin extracted from the zea-xanthin-accumulating *E. coli* strain containing the plasmid pACCAR25ΔcrtX [35].

### 2.4. Extraction of Genomic DNA from N. spongiae

Genomic DNA was extracted from approximately 10 mg of pellets of *N. spongiae* using NucleoSpin Tissue (Takara Bio Inc., Shiga, Japan), according to the manufacturer’s instructions. For the elution of genomic DNA, 0.1 mL of an elution buffer was used.

### 2.5. Cloning of the Carotenogenic Gene Cluster from N. spongiae

Seven carotenogenic genes, *crtI*, *crtB*, *crtZ*, *crtD*, *cruF*, *crtY*, and *crtA-OH*, formed a carotenogenesis cluster and were amplified as a single DNA fragment by PCR from *N. spongiae* genomic DNA using KOD-Plus polymerase (Toyobo, Osaka, Japan) and primers MyxClu_Fw1 and MyxClu_Rv1. The amplified DNA fragment was inserted into the EcoRI and HindIII restriction sites of the pET21a vector (Merck KGaA, Darmstadt, Germany) by an infusion cloning reaction following the manufacturer’s protocol (Takara Bio Inc.), resulting in the plasmid, pET21-MyxClu. The primer sequences are listed in Table S1.

### 2.6. Functional Analysis of cruF, crtY, crtD, crtA-OH, and crtZ Genes from N. spongiae

DNA fragments containing genes from the carotenogenic gene cluster of *N. spongiae*, *cruF*, *crtY*, *crtD*, *crtA-OH*, and *crtZ* were amplified alone or in combination by PCR and inserted into two multiple cloning sites (MCS1 and MCS2) of the pETDuet-1 vector (Merck KGaA), as shown in Figure S1. These plasmids were introduced into *E. coli* strain JM109 along with pACCRT-EIB, which contained the three carotenoid biosynthesis genes *crtE*, *crtB*, and *crtI* from *Pantoea ananatis* [35]. The transformants were cultured at 37 °C in 100 mL of a 2YT medium containing chloramphenicol (15 mg L^−1^) and ampicillin (50 mg L^−1^) until the OD_600_ reached 0.4–0.6. The transformants were then treated with 0.05 mM isopropyl-β-D-thiogalactopyranoside (IPTG), followed by culture at 25 °C with 200 rpm for 48 h.

### 2.7. Reconstruction of Plasmid for Myxol Production

Two DNA fragments from the carotenogenic gene cluster of *N. spongiae*, one containing *crtI*, *crtB*, *crtZ*, *crtD*, *cruF*, and *crtY* and the other containing *crtA-OH*, were amplified by PCR using KOD-Plus polymerase and the primers MyxClu_Fw2, MyxClu_Rv2, crtAOH_Fw1, and crtAOH_Rv1, respectively. Additionally, *crtE*, located at a different location from the gene cluster, was amplified using primers crtE_Fw1 and crtE_Rv1. The three DNA fragments were simultaneously inserted into the BamHI and NotI restriction sites of the pACYCDuet-1 vector (Merck KGaA) using an infusion cloning reaction following the manufacturer’s protocol, resulting in plasmid pACYC-MyxClu-E. The plasmid was introduced into *E. coli* strain BL21, and the transformants were cultured in 100 mL of a 2YT medium containing chloramphenicol (30 mg L^−1^) at 37 °C until the OD_600_ reached 0.4–0.6. Transformants were then treated with 0.05 mM IPTG, followed by culture at 25 °C with 200 rpm for 48 h.

### 2.8. Extraction and Analysis of Carotenoids from E. coli Cells

Carotenoids in recombinant *E. coli* cell pellets were extracted and identified as described above in *N. spongiae.*

## 3. Results and Discussion

### 3.1. Analysis of Carotenogenic Gene Cluster in Nonlabens

**Figure 1:**
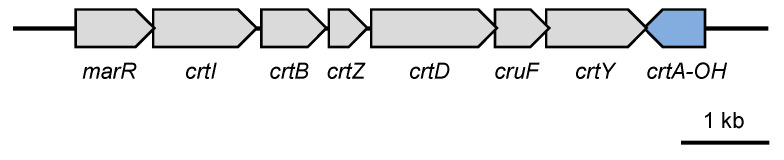
Genetic organization of genes in myxol biosynthetic gene cluster in marine flavobacteria *Nonlabens spongiae* JCM 13191^T^. Gray and blue arrows indicate candidate genes encoding MarR family transcriptional regulator (MerR), phytoene desaturase (CrtI), phytoene synthase (CrtB), carotene hydroxylase (CrtZ), carotenoid 3,4-desaturase (CrtD), carotenoid 1,2-hydoratase (CruF), lycopene cyclase (CrtY), and carotenoid 2′-hydroxylase (CrtA-OH), which are responsible for myxol and/or zeaxanthin production. Arrows indicate direction of transcription.

The orange colony color of the marine flavobacteria *Nonlabens spongiae* JCM 13191^T^ suggested the presence of carotenoid pigment. By performing a homologous search for carotenoid biosynthesis genes in the genome, we identified a carotenoid biosynthesis gene cluster (Figure 1) with seven candidate genes highly homologous to the functional enzymes (Table 1).

The first candidate gene encoded the MerR family of transcription factors. Other genes had high sequence homology to functional enzymes: *crtB*, phytoene synthase from *Pantoea ananatis* [7]; *crtI*, phytoene desaturase from *Flavobacterium* sp. strain P99-3 [16]; *crtY*, lycopene cyclase from P99-3 [16]; *crtD*, carotene 3.4-desaturase from P99-3 [18]; and crtZ, β-carotene hydroxylase from P99-3 [17]. One ORF was highly homologous to *cruF*, carotenoid 1,2-hydratase (ψ-end group) from *Planococcus maritimus* strain iso-3 [36], but not to the same functional enzyme *crtC*, carotenoid 1,2-hydratase (ψ-end group) from *Rhodobacter capsulatus* [5,6]. One ORF showed high homology to *crtA*, a spheroidene monooxygenase from *Rhodobacter capsulatus* [6]; however, this carotenoid did not contain a keto group. It also had high homology to *crtA-OH*, a carotenoid 2-hydroxylase from P99-3 [19], suggesting that it might be the same enzyme. Additionally, *crtE*, a geranylgeranyl pyrophosphate synthase that was highly homologous to *P. ananatis* [7], was located outside the cluster. Consequently, homology searches of genomic DNA sequence data revealed candidate genes for carotenoid biosynthesis, and the characteristics of the enzymes encoded by these candidate genes predicted that *N. spongiae* could potentially produce myxol and/or zeaxanthin.

Additionally, we found that eight other species of genus *Nonlabens* including *N. dokdonensis* DSW-6^T^, *N. xylanidelens* DSM16809^T^, *N. tegetincola* JCM 12886^T^ and NBRC 100970, *N. arenilitoris* KCTC 32109^T^, *N. ulvanivorans* PLR^T^, *N. agnitus* JCM 17109^T^, and *N. marinus* S1-08^T^ had the genes in carotenoid biosynthesis gene clusters in the same order. 

The marine *Flavobacterium* sp. strain P99-3 has been reported to produce myxol [15], containing a gene cluster similar to *Nonlabens*. The 16S rRNA sequence (AB106141.1) [16] showed 99.58% homology to that of *N. tegetincola* 12886^T^ (AY987349.1), suggesting that this strain might be one strain of this species [24].

### 3.2. Identification of Carotenoids from N. spongiae

**Figure 2:**
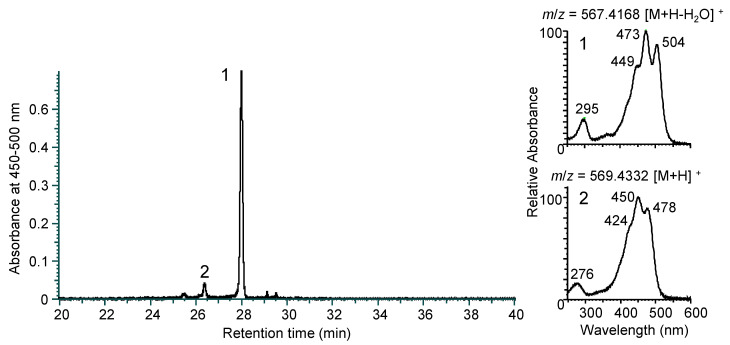
The LC-MS analysis of carotenoids extracted from *Nonlabens spongiae* JCM 13191^T^. The HPLC chromatogram is shown on the left, and the PDA spectra and LC-MS molecular ions for each peak are shown on the right. MS spectra are shown in Figure S2. Peak 1, myxol; peak 2, zeaxanthin.

*N. spongiae* JCM 13191^T^ produces orange pigment. Following the organic solvent extraction of the pigments, an LC-MS analysis revealed two pigment peaks (Figure 2 and Figure S2). The retention time of peak 1 was 28.0 min with absorption maxima at 295, 449, 473, and 504 nm in the HPLC eluent. The positive-ion high-resolution mass spectrum detected a molecular ion at *m*/*z* 567.4168 ([M+H-H_2_O]^+^, calculated as 567.4197 for C_40_H_55_O_2_), corresponding to myxol. The retention times of peak 2 was 26.3 min with absorption maxima at 276, 424, 450, and 478 nm. The positive-ion high-resolution mass spectrum detected a molecular ion at *m*/*z* 569.4332 ([M+H]^+^, calculated as 569.4353 for C_40_H_57_O_2_), corresponding to zeaxanthin. Thus, two carotenoids were identified, and they were predicted from the gene cluster same as above.

Furthermore, the HPLC retention times and absorption spectra of two peaks corresponded to those of the carotenoids from *N. marinus* S1-08^T^, identified as (3*R*,2′*S*)-myxol and (3*R*,3′*R*)-zeaxanthin using ^1^H-NMR and CD spectroscopic data, respectively [37], also including that of chemically synthesized (3*R*,2′*S*)-myxol [34]. Thus, the two carotenoids in *N. spongiae* were identified as myxol and zeaxanthin. Myxol comprised approximately 90 mol% of the total carotenoids. *N. marinus* S1-08^T^ produced both myxol (40 mol%) and zeaxanthin (58 mol%), whereas *N. arenilitoris* KCTC 32109^T^ produced mainly myxol (97 mol%) and trace amounts of zeaxanthin (3 mol%). The compositions of myxol and zeaxanthin varied depending on the species, culture conditions, and/or enzyme characteristics.

### 3.3. Functional Analysis of Carotenogenic Genes in Cluster from N. spongiae

**Figure 3:**
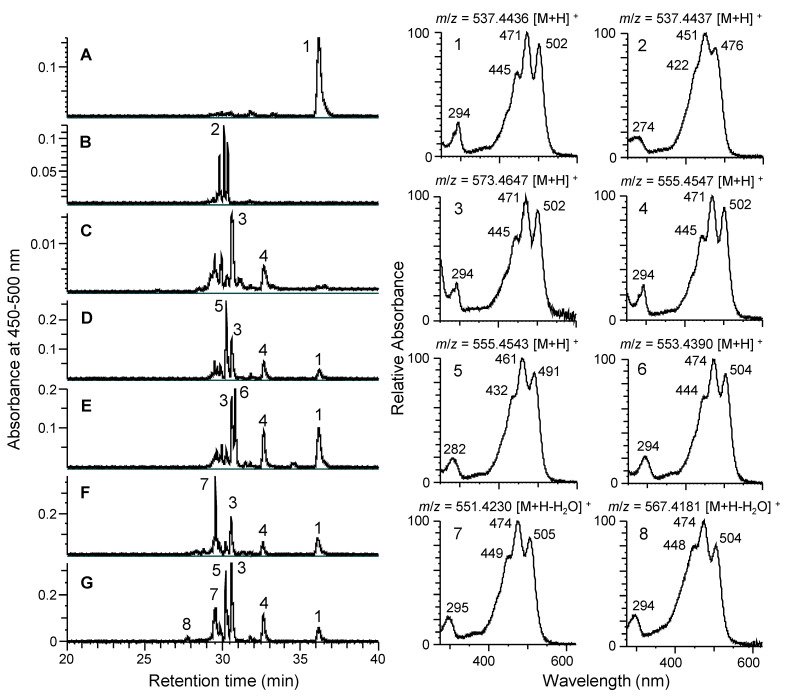
The LC-MS analysis of the carotenoids produced by the *E. coli* cells harboring different combinations of plasmids. The HPLC chromatograms are shown on the left, and the PDA spectra and LC-MS molecular ions for each peak are shown on the right. MS spectra are shown in Figure S3. (**A**) pACCRT-EIB, encoding *crtE*, *crtB*, and *crtI* from *P. ananatis* and pETDuet1 (the empty vector); (**B**) pACCRT-EIB and pETDuet-Y; (**C**) pACCRT-EIB and pETDuet-F; (**D**) pACCRT-EIB and pETDuet-FY; (**E**) pACCRT-EIB and pETDuet-FYD; (**F**) pACCRT-EIB and pETDuet-FYDA; (**G**) pACCRT-EIB and pETDuet-FYDAZ. Peak 1, lycopene; peak 2, β-carotene; peak 3, dihydroxylycopene; peak 4, hydroxylycopene; peak 5, 1′-hydroxy-γ-carotene; peak 6, 1′-hydroxytorulene; peak 7, deoxy-myxol; and peak 8, myxol.

We used a heterologous *E. coli* expression system to investigate the functions of genes in the carotenogenic gene cluster from *N. spongiae* [38]. The carotenoids were characterized using LC-MS, and their HPLC retention times, UV–visible absorption spectra, and molecular masses (Figure 3 and Figure S3) were compared with those of chemically synthesized carotenoids [34] and previous reports [18]. The *E. coli* strain expressing plasmid pACCRT-EIB containing *crtE*, *crtB*, and *crtI* from *P. ananatis* accumulated lycopene (peak 1 in Figure 3A) [35]. When *crtY* from *N. spongiae* was introduced alone into the lycopene-accumulating *E. coli*, β-carotene (peak 2 in Figure 3B) and its isomers were produced, whereas when *cruF* from *N. spongiae* was introduced alone, dihydroxylycopene (peak 3 in Figure 3C) and hydroxylycopene (peak 4 in Figure 3C) were mainly produced. In contrast, when both *crtY* and *cruF* were introduced simultaneously, 1′-hydroxy-γ-carotene (peak 5 in Figure 3D) was mainly produced. These results demonstrated that the CrtY and CruF of *N. spongiae* could catalyze the cyclization and hydration reactions using not only lycopene but also hydroxylycopene or γ-carotene as substrates, respectively.

Although the deduced amino acid sequence homology of the *crtY* of *N. spongiae* is higher in the lycopene β-monocyclase gene (*crtYm*) of *Flavobacterium* P99-3 (47% amino acid identity) than in the lycopene β-bicyclase gene (*crtY*) of *P. ananatis* (26% amino acid identity) [16], introducing the *crtY* from *N. spongiae* alone into the lycopene-accumulating *E. coli* strain resulted in the primary synthesis of the bicyclic carotenoid, β-carotene, from lycopene, with only a small amount of the monocyclic carotenoid, γ-carotene, detected. In contrast, when both *crtY* and *cruF* were introduced simultaneously, the monocyclic carotenoid 1′-hydroxy-γ-carotene (peak 5 in Figure 3D) was mainly produced, suggesting that the CrtY and/or CruF of *N. spongiae* may have different affinities for their substrates.

In cyanobacteria, the CruF gene was identified as a C-1′,2′-hydratase, and was required for myxol biosynthesis by *Synechococcus* sp. strain PCC 7002 [20]. Sun et al. (2009) identified the *cruF* gene, *dr0091* and *dgeo2309*, in nonphotosynthetic bacteria *Deinococcus radiodurans* R1 and *Deinococcus geothermalis* DSM 11300, respectively, and demonstrated that they were responsible for the C-1′,2′-hydration of γ-carotene in the deinoxanthin biosynthetic pathway [39]. Moreover, genes encoding CrtC-type carotenoid 1,2-hydratase have been found in purple bacteria such as *Rhodobacter capsulatus* [5], *Rubrivivax gelatinosus* [40], and *Thiocapsa roseopersicina* [41], which are involved in the biosynthesis of spirilloxanthin and spheroidene. This study’s results provide the first evidence that CruF is a carotenoid 1,2-hydratase involved in the myxol biosynthetic pathway in *Flavobacterium* species. Two types of carotenoid 1,2-hydratases, CruF and CrtC, were functionally confirmed, and no amino acid sequence homology was observed between these two proteins. The distribution of these two enzymes was particularly interesting.

In addition to *crtY* and *cruF*, when *crtD* from *N. spongiae* was introduced into the lycopene-accumulating *E. coli* strain, 1′-hydroxytorulene (peak 6 in Figure 3E) was mainly produced. These results indicated that CrtD from *N. spongiae* could catalyze the desaturation reaction of 1′-hydroxy-γ-carotene.

CrtA, a spheroidene monooxygenase found only in purple bacteria, introduces a keto group at C-2 of the ψ-end group [3,5]. A protein highly homologous to CrtA was found in *Flavobacterium* P99-3, which introduces a hydroxyl group at C-2 of the ψ-end group and is called CrtA-OH [19]. When crtA-OH of *N. spongiae* was introduced into the lycopene-accumulating *E. coli* strain in addition to *crtY*, *cruF*, and *crtD*, 1′-hydroxytorulene was converted to deoxymyxol (peak 7 in Figure 3F). Furthermore, *crtZ* was introduced into the above *E. coli* strain; deoxymyxol was converted into myxol (peak 8 in Figure 3G). Choi et al. (2006) reported that CrtZ of P99-3 has dicyclic carotenoid 3,3′-hydroxylase activity to produce astaxanthin and zeaxanthin from canthaxanthin and β-carotene, respectively [17]. The findings of this study provide the first report to directly indicate that the CrtA-OH and CrtZ of *N. spongiae* catalyze the hydroxylation reaction from 1′-hydroxytorulene to deoxymyxol and then to myxol.

**Figure 4:**
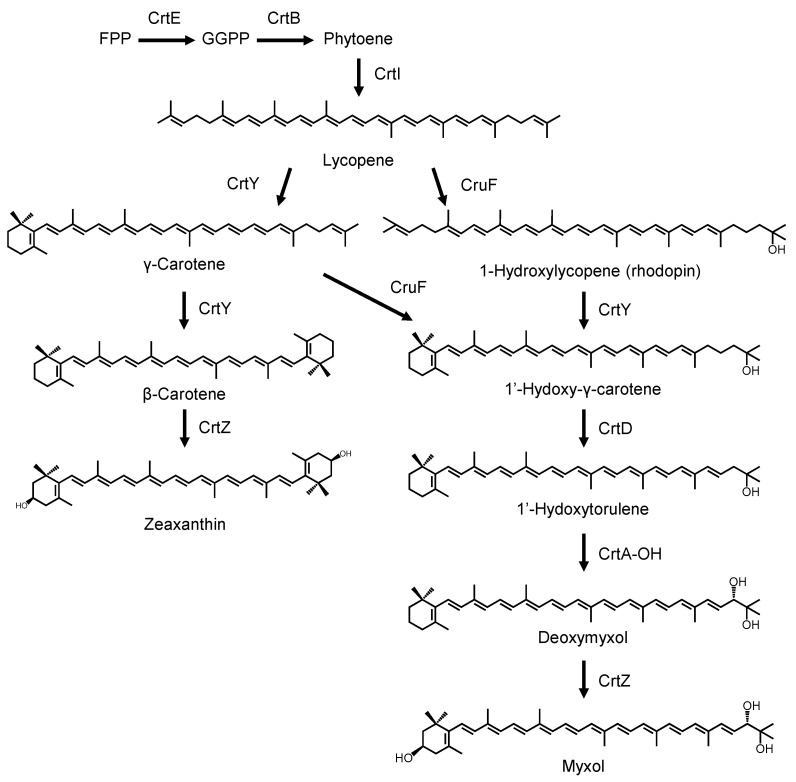
Proposed biosynthetic pathway of myxol in marine flavobacteria *Nonlabens spongiae* JCM 13191^T^. CrtE, geranylgeranyl pyrophosphate synthase; CrtB, phytoene synthase; CrtI, phytoene desaturase; CrtY, lycopene cyclase; CruF, carotenoid 1,2-hydoratase; CrtD, carotenoid 3,4-desaturase; CrtA-OH, carotenoid 2′-hydroxylase; CrtZ, β-carotene hydroxylase; FPP, farnesyl pyrophosphate; GGPP, geranylgeranyl pyrophosphate.

We also confirmed that *crtB* and *crtI* in the gene cluster from *N. spongiae* were responsible for lycopene biosynthesis. Thus, the functions of all carotenogenesis genes except for *crtE* in *N. spongiae* were functionally identified. Based on these results, we propose a biosynthetic pathway for myxol production in *N. spongiae* as shown in Figure 4.

### 3.4. Production of Myxol Using Reconstructed Gene Cluster and crtE from N. spongiae in E. coli

The production of myxol in *E. coli* requires a geranylgeranyl pyrophosphate synthase (CrtE) gene in addition to the seven enzyme genes (*crtB*, *crtI*, *crtY*, *cruF*, *crtD*, *crtA-OH*, and *crtZ*) found in the cluster of *N. spongiae*. Using the genome sequence of *N. spongiae*, we identified a candidate *crtE* at a locus different from that of the gene cluster. To investigate the functions of the *crtE*-like gene, it was transformed into the *E. coli* strain carrying the plasmid pACCAR25ΔcrtE, which contained all genes required for the production of zeaxanthin diglucoside except *crtE*. *E. coli* transformants produced zeaxanthin, zeaxanthin monoglucoside, and zeaxanthin diglucoside, indicating that the *crtE* gene product from *N. spongiae* encodes geranylgeranyl pyrophosphate synthase.

**Figure 5:**
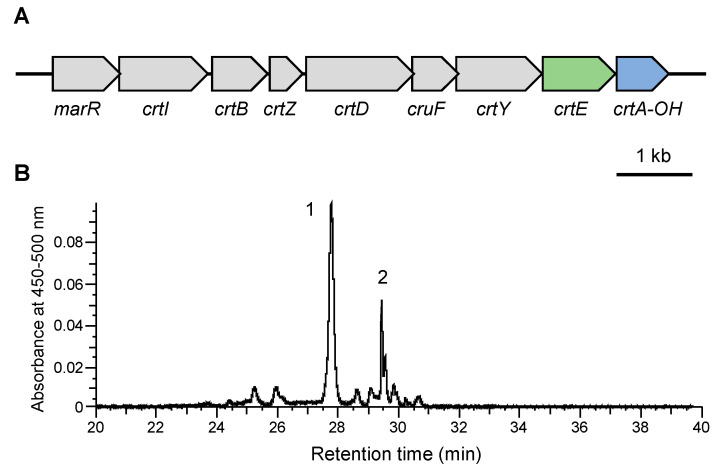
The production of myxol using the reconstructed gene cluster from *Nonlabens spongiae* JCM 13191^T^ in *E. coli*. (**A**) Structure of the plasmid, pACYC-MyxClu-E, used for the production of myxol in *E. coli*. Gray arrows indicate the cluster genes in *N. spongiae* described in Figure 1. The blue arrow indicates the *crtA-OH*, which was excised from the cluster and subsequently reconnected in the reverse direction. The green arrow indicates the geranylgeranyl pyrophosphate synthase (CrtE) gene, located away from the cluster in the *N. spongiae* genome. (**B**) The LC-MS analysis of carotenoids produced by the *E. coli* cells harboring pACYC-MyxClu-E. Peak 1, myxol; peak 2, 1′-hydroxy-γ-carotene.

To efficiently produce myxol in *E. coli*, the carotenoid biosynthesis gene cluster from *N. spongiae* was reconstructed by introducing the isolated *crtE* and reversing the orientation of *crtA-OH*, ensuring that all genes were transcribed in the same direction (Figure 5A). When this reconstructed gene cluster was introduced into *E. coli*, myxol predominantly accumulated (peak 1 in Figure 5B). The productivity of myxol was approximately 400 mg L^−1^ when the recombinant *E. coli* was cultured for 48 h after IPTG induction with 100 mL of a medium. This study is the first report for the successful production of myxol in *E. coli* by introducing a single DNA fragment consisting of myxol biosynthesis genes.

*E. coli* is an excellent host for the production of various carotenoids, such as lycopene, β-carotene, zeaxanthin, and astaxanthin, using metabolic engineering techniques [42,43]. The introduction of isopentenyl diphosphate isomerase (*idi*), 1-deoxy-D-xylulose 5-phosphate synthase (*dxs*), and 1-deoxy-D-xylulose 5-phosphate reductoisomerase (*dxr*) in the non-mevalonate (MEP) pathway genes of *Saccharomyces cerevisiae* and *Haematococcus pluvialis* into *E. coli* improves carotenoid productivity [44,45]. In contrast, the introduction of heterologous mevalonate (MVA) pathway genes encoding 3-hydroxy-3-methylglutaryl CoA (HMGCoA) synthase, HMG-CoA reductase, MVA kinase, phosphormevalonate (PMVA) kinase, and diphosphomevalonate (DPMVA) decarboxylase, and the *idi* type 2 gene, into *E. coli* enhanced carotenoid productivity [46]. Additionally, it has been reported that heterologous carotenoid formation was strongly dependent on *E. coli* strains [23,47]. To achieve enhanced productivity of rare myxol in the future, it is necessary to introduce the MEP pathway genes and/or MVA pathway genes into myxol-producing *E. coli* and to select an optimal host strain.

## Figures and Tables

**Table 1:** Candidate carotenoid biosynthesis genes in *Nonlabens spongiae* JCM1391^T^.

Gene	Enzyme	Query Sequence for BLASTP		*Nonlabens spongiae* JCM13191^T^	
		Accession No.	Organisms	Accession No.	Identity (%) /e-Value
*crtE*	Geranylgeranyl pyrophosphate synthase	BAA14124	*Pantoea ananatis* [7]	WP_085767001	30/3e-22
*crtB*	Phytoene synthase	BAA14128	*Pantoea ananatis* [7]	WP_085765367	26/2e-17
*crtI*	Phytoene desaturase	BAC77668	*Flavobacterium* P99-3 [16]	WP_085765368	83/0.0
*crtY*	Lycopene cyclase	BAC77673	*Flavobacterium* P99-3 [16]	WP_085765363	47/6e-124
*cruF*	Carotenoid 1,2-hydratase	BCT98105	*Planococcus maritimus* [36]	WP_085765364	36/1e-17
*crtD*	Carotenoid 3,4-desaturase	BAC77671	*Flavobacterium* P99-3 [18]	WP_085765365	73/0.0
*crtA-OH*	Carotenoid 2-hydroxylase	BAC77674	*Flavobacterium* P99-3 [19]	WP_085765362	50/2e-81
*crtA*	Spheroidene monooxygenase	CAA77539	*Rhodobacter capsulatus* [6]	WP_085765362	31/2e-30
*crtZ*	β-Carotene hydroxylase	BAC77670	*Flavobacterium* P99-3 [17]	WP_085765366	80/1e-79

Query sequences are selected based on functional enzymes.

## Data Availability

The original contributions presented in the study are included in the article, further inquiries can be directed to the corresponding author.

## Supplementary Materials

The following supporting information can be downloaded at: https://www.mdpi.com/article/10.3390/genes16020202/s1, Table S1: List of primers used in this study.; Figure S1: Construction of plasmids for functional analysis of each putative myxol biosynthetic gene in *Nonlabens spongiae* JCM13191^T^; Figure S2: MS spectrum of peaks 1 and 2 in Figure 2; Figure S3: MS spectrum of peaks 1–8 in Figure 3.

## Abbreviations

The following abbreviations are used in this manuscript:

APCI	atmospheric-pressure chemical ionization
FPP	farnesyl diphosphate
JCM	Japan Collection of Microorganisms

## References

[1] Frank H.A., Cogdell R.J. (1996). Carotenoids in photosynthesis. Photochem. Photobiol..

[2] Paulsen H., Frank H.A., Young A.J., Britton G., Cogdell R.J. (1999). Carotenoids and the assembly of light-harvesting complexes. The Photochemistry of Carotenoids.

[3] Takaichi S., Ramawat K.G., Merillon J.M. (2025). Carotenoids in carotenogenic organisms: Distribution, biosynthesis, and functions. Natural Products: Phytochemistry, Botany and Metabolism of Alkaloids, Phenolics and Terpenes.

[4] Maoka T. (2020). Carotenoids as natural functional pigments. J. Nat. Med..

[5] Armstrong G.A., Alberti M., Leach F., Hearst J.E. (1989). Nucleotide sequence, organization, and nature of the protein products of the carotenoid biosynthesis gene cluster of *Rhodobacter capsulatus*. Mol. Gen. Genet..

[6] Gerjets T., Steiger S., Sandmann G. (2009). Catalytic properties of the expressed acyclic carotenoid 2-ketolases from *Rhodobacter capsulatus* and *Rubrivivax gelatinosus*. Biochim. Biophys. Acta.

[7] Misawa N., Nakagawa M., Kobayashi K., Yamano S., Izawa Y., Nakamura K., Harashima K. (1990). Elucidation of pathway the *Erwinia uredovora* carotenoid biosynthetic by functional analysis of gene products expressed in *Escherichia coli*. J. Bacteriol..

[8] Hannibal L., Lorquin J., D’ortoli N.A., Garcia N., Chaintreuil C., Masson-Boivin C., Dreyfus B. (2000). Isolation and characterization of canthaxanthin biosynthesis genes from the photosynthetic bacterium *Bradyrhizobium* sp. strain ORS278. J. Bacteriol..

[9] Giraud E., Hannibal L., Fardoux J., Jaubert M., Jourand P., Dreyfus B., Sturgis J.N., Verméglio A. (2004). Two distinct crt gene clusters for two different functional classes of carotenoid in *Bradyrhizobium*. J. Biol. Chem..

[10] Kang M., Chhetri G., Kim J., Kim I., Seo T. (2021). *Sphingomonas sabuli* sp. nov., a carotenoid-producing bacterium isolated from beach sand. Int. J. Syst. Evol. Microbiol..

[11] Serrano S., Mendo S., Caetano T. (2022). Haloarchaea have a high genomic diversity for the biosynthesis of carotenoids of biotechnological interest. Res. Microbiol..

[12] Hertzberg S., Liaaen-Jensen S. (1969). The structure of myxoxanthophyll. Phytochemistry.

[13] Takaichi S., Mochimaru M. (2007). Carotenoids and carotenogenesis in cyanobacteria: Unique ketocarotenoids and carotenoid glycosides. Cell Mol. Life Sci..

[14] Sugiyama K., Ebisawa M., Yamada M., Nagashima Y., Suzuki H., Maoka T., Takaichi S. (2017). Functional lycopene cyclase (CruA) in cyanobacterium, *Arthrospira platensis* NIES-39, and its role in carotenoid synthesis. Plant Cell Physiol..

[15] Yokoyama A., Miki W. (1995). Isolation of myxol from a marine bacterium *Flavobacterium* sp. associated with a marine sponge. Fish. Sci..

[16] Teramoto M., Takaichi S., Inomata Y., Ikenaga H., Misawa N. (2003). Structural and functional analysis of a lycopene L-monocyclase gene isolated from a unique marine bacterium that produces myxol. FEBS Lett..

[17] Choi S.K., Matsuda S., Hoshino T., Peng X., Misawa N. (2006). Characterization of bacterial β-carotene 3,3′-hydroxylases, CrtZ, and P450 in astaxanthin biosynthetic pathway and adonirubin production by gene combination in *Escherichia coli*. Appl. Microbiol. Biotechnol..

[18] Teramoto M., Rählert N., Misaswa N., Sandmann G. (2004). 1-Hydroxy monocyclic carotenoid 3,4-dehydrogenase from a marine bacterium that produces myxol. FEBS Lett..

[19] Rählert N., Fraser P.D., Sandmann G. (2009). A crtA-related gene from *Flavobacterium* P99-3 encodes a novel carotenoid 2-hydroxylase involved in myxol biosynthesis. FEBS Lett..

[20] Graham J.E., Bryant D.A. (2009). The biosynthetic pathway for myxol-2 fucoside (myxoxanthophyll) in the cyanobacterium *Synechococcus* sp. strain PCC 7002. J. Bacteriol..

[21] Umeno D., Arnold F.H. (2004). Evolution of a pathway to novel long-chain carotenoids. J. Bacteriol..

[22] Li L., Furubayashi M., Wang S., Maoka T., Kawai-Noma S., Saito K., Umeno D. (2019). Genetically engineered biosynthetic pathways for nonnatural C_60_ carotenoids using C_5_-elongases and C_50_-cyclases in *Escherichia coli*. Sci. Rep..

[23] Takemura M., Kudo A., Higuchi Y., Maoka T., Sahara T., Yaoi K., Ohdan K., Umeno D., Misawa N. (2019). Pathway engineering for efficient biosynthesis of violaxanthin in *Escherichia coli*. Appl. Microbiol. Biotechnol..

[24] Lau S.C.K., Tsoi M.M.Y., Li X., Plakhotnikova I., Dobretsov S., Wu M., Wong P.K., Pawlik J.R., Qian P.Y. (2006). *Stenothermobacter spongiae* gen. nov., sp. nov., a novel member of the family *Flavobacteriaceae* isolated from a marine sponge in the Bahamas, and emended description of *Nonlabens tegetincola*. Int. J. Syts. Evol. Microbiol..

[25] Yi H., Chun J. (2012). Unification of the genera *Nonlabens*, *Persicivirga*, *Sandarakinotalea* and *Stenothermobacter* into a single emended genus, *Nonlabens*, and description of *Nonlabens agnitus* sp. nov. Syst. Appl. Microbiol..

[26] Kwon S.K., Kim B.K., Song J.Y., Kwak M.J., Lee C.H., Yoon J.H., Oh T.K., Kim J.F. (2013). Genomic makeup of the marine flavobacterium *Nonlabens* (*Donghaeana*) *dokdonensis* and identification of a novel class of Rhodopsins. Genome Biol. Evol..

[27] Kopel M., Helbert W., Henrissat B., Doniger T., Banin E. (2014). Draft genome sequence of *Nonlabens ulvanivorans*, an Ulvan-Degrading Bacterium. Genome Announc..

[28] Kumagai Y., Yoshizawa S., Nakajima Y., Watanabe M., Fukunaga T., Ogura Y., Hayashi T., Oshima K., Hattori M., Ikeuchi M. (2018). Solar-panel and parasol strategies shape the proteorhodopsin distribution pattern in marine Flavobacteriia. ISME J..

[29] Seo Y.L., Jung J., Song C., Kwon Y.M., Jung H.S., Eyun S., Jeon C.O. (2021). *Nonlabens ponticola* sp. nov., isolated from seawater and reclassification of *Nonlabens sediminis* as a later heterotypic synonym of *Nonlabens tegetincola*. Int. J. Syst. Evol. Microbiol..

[30] Khan S.T., Nakagawa Y., Harayama S. (2006). *Sandarakinotalea sediminis* gen. nov., sp. nov., a novel member of the family *Flavobacteriaceae*. Int. J. Syts. Evol. Microbiol..

[31] Lau S.C.K., Tsoi M.M.Y., Li X., Plakhotnikova I., Dobretsov S., Wong P.K., Pawlik J.R., Qian P.Y. (2005). *Nonlabens tegetincola* gen. nov., sp. nov., a novel member of the family *Flavobacteriaceae* isolated from amicrobial mat in a subtropical estuary. Int. J. Syts. Evol. Microbiol..

[32] Park S., Kang C.H., Yoon J.H. (2013). *Nonlabens arenilitoris* sp. nov., a member of the family *Flavobacteriaceae* isolated from seashore sand. Antonie Leeuwenhoek.

[33] O’Sullivan L.A., Rinna J., Humphreys G., Weightman A.J., Fry J.C. (2006). Culturable phylogenetic diversity of the phylum ‘*Bacteroidetes*’ from river epilithon and coastal water and description of novel members of the family *Flavobacteriaceae*: *Epilithonimonas tenax* gen. nov., sp. nov. and *Persicivirga xylanidelens* gen. nov., sp. nov. Int. J. Syts. Evol. Microbiol..

[34] Yamano Y., Masumoto M., Takaichi S., Wada A. (2018). Total synthesis of myxol and deoxymyxol stereoisomers and their application to determining the absolute configurations of the natural products. Tetrahedron.

[35] Misawa N., Sato Y., Kondo K., Yokoyama A., Kajiwara S., Saito T., Ohtani T., Miki W. (1995). Structure and functional analysis of a marine bacterial carotenoid biosynthesis gene cluster and astaxanthin biosynthetic pathway proposed at the gene level. J. Bacteriol..

[36] Takemura M., Takagi C., Aikawa M., Araki K., Choi S.K., Itaya M., Shindo K., Misawa N. (2021). Heterologous production of novel and rare C_30_-carotenoids using *Planococcus* carotenoid biosynthesis genes. Microb. Cell Fact..

[37] Fujiwara T., Hosaka T., Hasegawa-Takano M., Nishimura Y., Tominaga K., Mori K., Nishino S., Takahashi Y., Uchikubo-Kamo T., Hanada K. (2024). Carotenoid pigments enhance rhodopsin-mediated phototrophy by light-harvesting and photocycle-accelerating. bioRxiv.

[38] Harada H., Misawa N. (2009). Novel approaches and achievements in biosynthesis of functional isoprenoids in *Escherichia coli*. Appl. Microbiol. Biotechnol..

[39] Sun Z., Shen S., Wang C., Wang H., Hu Y., Jiao J., Ma T., Tian B., Hua Y. (2009). A novel carotenoid 1,2-hydratase (CruF) from two species of the non-photosynthetic bacterium *Deinococcus*. Microbiology.

[40] Ouchane S., Picaud M., Vernotte C., Reiss-Husson F., Astier C. (1997). Pleiotropic effects of *puf* interposon mutagenesis on carotenoid biosynthesis in *Rubrivivax gelatinosus*. J. Biol. Chem..

[41] Kovács Á.T., Rákhely G., Kovács K.L. (2003). Genes involved in the biosynthesis of photosynthetic pigments in the purple sulfur photosynthetic bacterium *Thiocapsa roseopersicina*. App. Environ. Microbiol..

[42] Misawa N., Maoka T., Takemura M. (2022). Carotenoids: Carotenoid and apocarotenoid analysis—Use of *E. coli* to produce carotenoid standards. Methods Enzymol..

[43] Wang C., Zhao S., Shao X., Park J.B., Jeong S.H., Park H.J., Kwak W.J., Wei G., Kim S.W. (2019). Challenges and tackles in metabolic engineering for microbial production of carotenoids. Microb. Cell Fact..

[44] Kajiwara S., Fraser P.D., Kondo K., Misawa N. (1997). Expression of an exogenous isopentenyl diphosphate isomerase gene enhances isoprenoid biosynthesis in *Escherichia coli*. Biochem. J..

[45] Albrecht M., Misawa N., Sandmann G. (1999). Metabolic engineering of the terpenoid biosynthetic pathway of *Escherichia coli* for production of the carotenoids β-carotene and zeaxanthin. Biotechnol. Lett..

[46] Harada H., Yu F., Okamoto S., Kuzuyama T., Utsumi R., Misawa N. (2009). Efficient synthesis of functional isoprenoids from acetoacetate through metabolic pathway-engineered *Escherichia coli*. Appl. Microbiol. Biotechnol..

[47] Chae H.S., Kim K.-H., Kim S.C., Lee P.C. (2010). Strain-dependent carotenoid productions in metabolically engineered *Escherichia coli*. Appl. Biochem. Biotechnol..

