# Supplementary material for: Product Speculation from Carotenogenic Gene Cluster of Nonlabens spongiae Genome, and Identification of Myxol and Functional Analysis of Each Gene
Source: Genes (Basel). 2025 Feb 7;16(2):202. doi: 10.3390/genes16020202 (PMC11855829; doi:10.3390/genes16020202)
Supplement: Supplementary file 1 [file genes-16-00202-s001.zip › genes-3453002-supplementary.pdf]

Supplementary Materials

Product speculation from the carotenogenic gene cluster of *Nonlabens spongiae* genome, and identification of myxol and functional analysis of each gene

Keisuke Nakazawa [1], Daiki Mineo [1], Takuya Harayama [1], Susumu Yoshizawa [2,3], Shinichi Takaichi [4] and Kenjiro Sugiyama [1,*]

[1] Department of Applied Chemistry, School of Advanced Engineering, Kogakuin University, Nakanomachi, Hachioji, Tokyo 192-0015, Japan
[2] Graduate School of Frontier Sciences, The University of Tokyo, Kashiwa, Chiba, 277-8563, Japan
[3] Atmosphere and Ocean Research Institute, The University of Tokyo, Kashiwa, Chiba, 277-8564, Japan
[4] Department of Molecular Microbiology, Faculty of Life Sciences, Tokyo University of Agriculture, Sakuragaoka, Setagaya, Tokyo, 156-8502, Japan

* Correspondence: bt13171@ns.kogakuin.ac.jp; Tel.: +81-426-28-4879

Table S1 List of primers used in this study

primer name	primer sequences (5' to 3')
MyxClu_Fw1	TCGCGGATCCGAATTCGTGGTTAAGAGTCGGTTTA
MyxClu_Rv1	GTGCGGCCGCAAGCTTATCACAACCGTTTCCGTTT
MyxClu_Fw2	ACCACAGCCAGGATCCGGTGGTTAAGAGTCGGTTTAG
MyxClu_Rv2	TCAGCCCTGGTTACGCCC
crtAOH_Fw1	ATGAATAGAATTAGTTAGATGTCAGAGCAAATCACAACC
crtAOH_Rv1	AAGCATTATGCGGCCGCTTACCTAAAATCTTCGGGCG
crtE_Fw1	CGTAACCAGGGCTGAATGGATTTACTCTCAGACTTAAAAAG
crtE_Rv1	CTAACTAATTCTATTCATCAACTGCAGAG
cruF_Fw1	AAGGAGATATACATATGATGATCAAAAAAGCGGCTCT
cruF_Rv1	TTACCAGACTCGAGGGTACCCTATGTCGTAATGATGAACC
crtY_Fw1	AAGGAGATATACATATGATGGTTCATCATTACGACATA
crtY_Rv1	TTACCAGACTCGAGGGTACCTCAGCCCTGGTTACGCCC
crtD_Fw1	AGAAGGAGATATACCATGAAAAAAGCTGCAGTCATAG
crtD_Rv1	TGTTCGACTTAAGCATCATGATTTCTCAATTAGATCAC
pmcs1_Fw	TGCTTAAGTCGAACAGAAAGTAA
pmcs1_Rv	GGTATATCTCCTTCTTAAAGTTAAACA
crtZ_Fw1	AGAAGGAGATATACCATGAGTACCCTCTTTTGGAT
crtD_Rv2	TCATGATTTCTCAATTAGATCAC
crtAOH_Fw2	TCTAATTGAGAAATCATGAATGTCAGAGCAAATCACAACC
crtAOH_Rv2	TGTTCGACTTAAGCATTACCTAAAATCTTCGGGCG
crtAOH_Fw3	TACATATGGCAGATCTATGTCAGAGCAAATCACAAC
crtAOH_Rv3	TTACCAGACTCGAGGGTACCTTACCTAAAATCTTCGGGCG

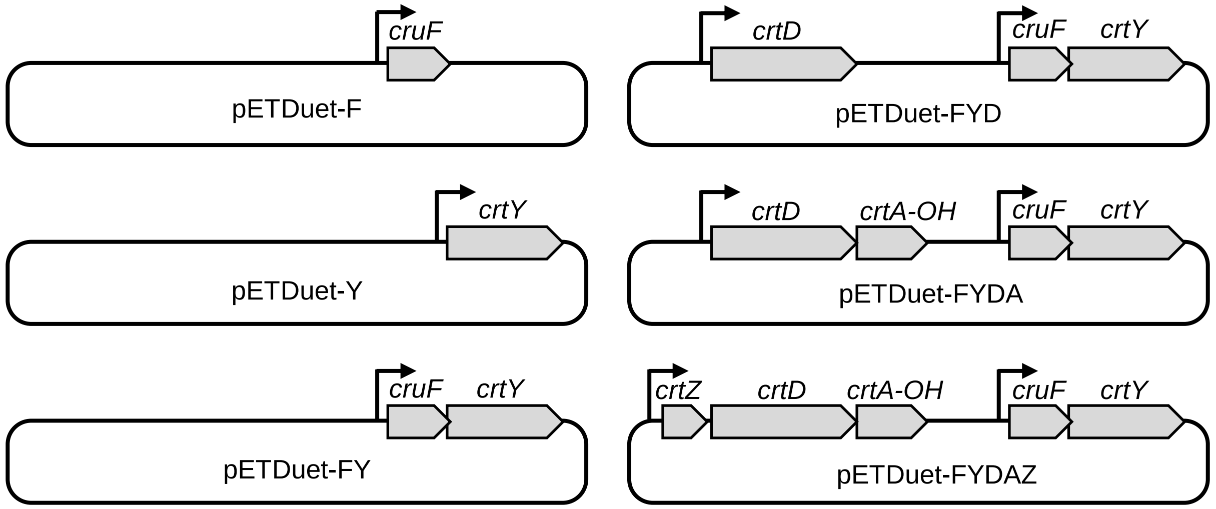

Figure S1 Construction of plasmids for functional analysis of each putative myxol biosynthetic genes in *Nonlabens spongiae* JCM13191^{T}.

pETDuet-F, which encodes *cruF* from *N. spongiae*; pETDuet-Y, which encodes *crtY* from *N. spongiae*; pETDuet-FY, which encodes *cruF* and *crtY* from *N. spongiae*; pETDuet-FYD, which encodes *cruF, crtY,* and *crtD* from *N. spongiae*; pETDuet-FYDA, which encodes *cruF, crtY, crtD*, and *crtA-OH* from *N. spongiae*; pETDuet-FYDAZ, which encodes *cruF, crtY, crtD*, *crtA-OH*, and *crtZ* from *N. spongiae.* Arrows indicate T7 promoter.

pETDuet-F: The *cruF* was amplified by PCR using primers cruF_Fw1 and cruF_Rv1 with pET21-MyxClu as a template, and inserted by infusion cloning reaction into the NdeI and KpnI restriction sites of pETDuet. **pETDuet-Y:** The *crtY* was amplified by PCR using primers crtY_Fw1 and crtY_Rv1 with pET21-MyxClu as a template, and inserted by infusion cloning reaction into the NdeI and KpnI restriction sites of pETDuet. **pETDuet-FY:** The *cruF* and *crtY* were amplified as one DNA fragment by PCR using primers cruF_Fw1 and crtY_Rv1 with pET21-MyxClu as a template, and inserted by infusion cloning reaction into the NdeI and KpnI restriction sites of pETDuet. **pETDuet-FYD:** The *crtD* was amplified by PCR using primers crtD_Fw1 and crtD_Rv1 with pET21-MyxClu as a template, and inserted by infusion cloning reaction into a linearized pETDuet-FY produced by inverse PCR using primers pmcs1_Fw and pmcs1_Rv. **pETDuet-FYDA:** The *crtD* and *crtA-OH* was amplified as one DNA fragment by PCR using primers crtD_Fw1 and crtA-OH_Rv2 with pETDuet-FYDAZ as a template, and inserted by infusion cloning reaction into a linearized pETDuet-FY produced by inverse PCR using primers pmcs1_Fw and pmcs1_Rv. **pETDuet-FYDAZ:** The *crtZ* and *crtD* was amplified as one DNA fragment by PCR using primers crtZ_Fw1 and crtD_Rv2 with pET21-MyxClu as a template, and the *crtA-OH* was amplified by PCR using primers crtA-OH_Fw2 and crtA-OH_Rv2 with pRSF-crtAOH as a template. The two DNA fragments were simultaneously inserted by infusion cloning reaction into a linearized pETDuet-FY produced by inverse PCR using primers pmcs1_Fw and pmcs1_Rv. pRSF-crtAOH: The *crtA-OH* was amplified by PCR from *N. spongiae* genomic DNA using primers crtA-OH_Fw3 and crtA-OH_Rv3, and inserted into the BglII and KpnI restriction sites of pRSFDuet-1 vector (Novagen).

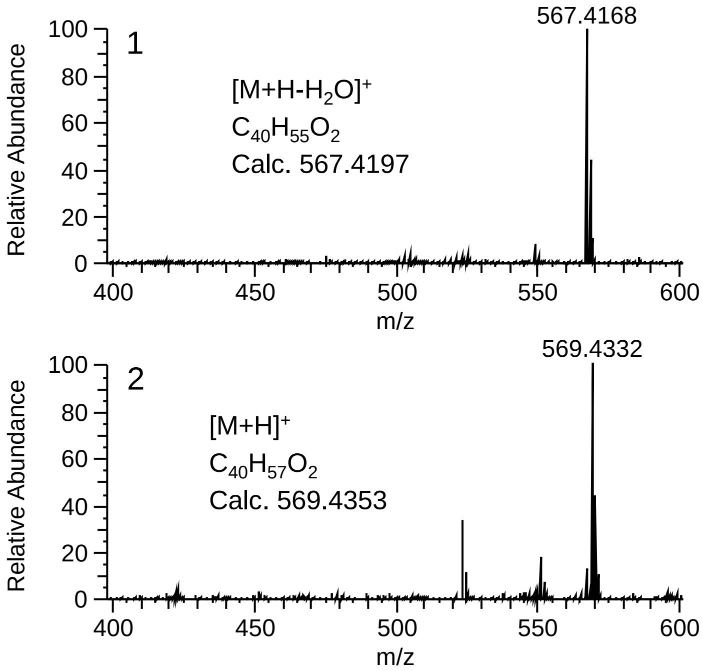

Figure S2 MS spectrum of peaks 1 and 2 in Figure 2. Calculated masses are shown inside. Peak 1, myxol; peak 2, zeaxanthin.

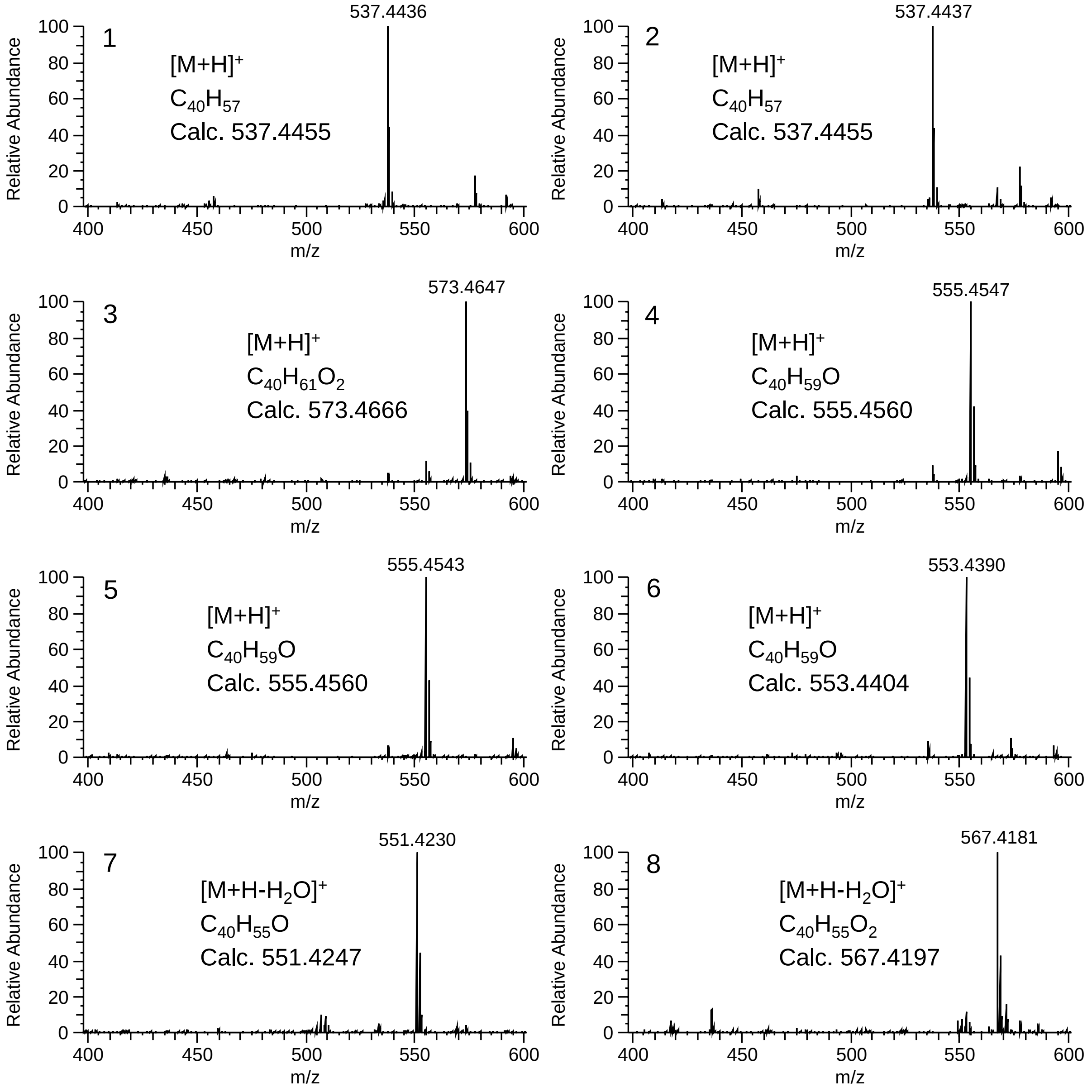

Figure S3 MS spectrum of peaks 1-8 in Figure 3. Calculated masses are shown inside. Peak 1, lycopene; peak 2, β-carotene; peak 3, dihydroxylycopene; peak 4, hydroxylycopene; peak 5, 1'-hydroxy-γ-carotene; peak 6, 1'-hydroxytorulene; peak 7, deoxymyxol; and peak 8, myxol.
